# One-year follow-up—case report of secondary tension pneumothorax in a COVID-19 pneumonia patient

**DOI:** 10.1007/s15010-021-01711-9

**Published:** 2021-10-08

**Authors:** Felix Endres, Judith Eva Spiro, Toki Anna Bolt, Amanda Tufman, Ben Ockert, Tobias Helfen, Fabian Gilbert, Boris M. Holzapfel, Wolfgang Böcker, Georg Siebenbürger

**Affiliations:** 1 Department of Orthopaedics and Trauma Surgery, Musculoskeletal University Center Munich (MUM), University Hospital, LMU Munich, Ziemssenstraße 5, 80336 Munich, Germany; 2 Department of Radiology, University Hospital, LMU Munich, Munich, Germany; 3 Department of Medicine V, University Hospital, Member of the German Center for Lung Research (DZL), LMU Munich, Munich, Germany

**Keywords:** COVID-19, Pneumothorax, Pneumonia, Multi-detector computed tomography, Viral infections

## Abstract

**Purpose:**

The Coronavirus Disease 2019 (COVID-19) may result not only in acute symptoms such as severe pneumonia, but also in persisting symptoms after months. Here we present a 1 year follow-up of a patient with a secondary tension pneumothorax due to COVID-19 pneumonia.

**Case presentation:**

In May 2020, a 47-year-old male was admitted to the emergency department with fever, dry cough, and sore throat as well as acute chest pain and shortness of breath. Sputum testing (polymerase chain reaction, PCR) and computed tomography (CT) confirmed infection with the severe acute respiratory syndrome coronavirus type 2 (SARS-CoV-2). Eleven days after discharge, the patient returned to the emergency department with pronounced dyspnoea after coughing. CT showed a right-sided tension pneumothorax, which was relieved by a chest drain (Buelau) via mini open thoracotomy. For a period of 3 months following resolution of the pneumothorax the patient complained of fatigue with mild joint pain and dyspnoea. After 1 year, the patient did not suffer from any persisting symptoms. The pulmonary function and blood parameters were normal, with the exception of slightly increased levels of D-Dimer. The CT scan revealed only discrete ground glass opacities (GGO) and subpleural linear opacities.

**Conclusion:**

Tension pneumothorax is a rare, severe complication of a SARS-CoV-2 infection but may resolve after treatment without negative long-term sequelae.

**Level of evidence:**

V.

## Background

The first infections with the severe acute respiratory syndrome coronavirus type 2 (SARS-CoV-2) were detected in humans in late 2019 [1]. While much is known about the acute symptoms of the Coronavirus Disease 2019 (COVID-19), long-term health effects of surviving patients remain unknown. Patients’ most common symptoms are fatigue, cognitive symptoms, dyspnoea, joint pain, and chest pain [2, 3].

In June 2020, we reported a case of a secondary tension pneumothorax as a complication of COVID-19 pneumonia [4]. Reports of pneumothorax as a complication of COVID-19 are rare and the reported pneumothoraces are often ventilator-induced [5, 6]. To our knowledge, a 1 year follow-up of a secondary tension pneumothorax due to COVID-19 infection has not been published so far.

## Case report

A 47-year-old male was admitted to our emergency department with dry cough, shortness of breath, and stenocardia. The symptoms had started 14 days before primary admission. Polymerase chain reaction (PCR) tests of nasal and pharyngeal swabs and sputum were positive for SARS-CoV-2-RNA N-gene 1, and negative for respiratory syncytial virus (RSV), Influenza-A and Influenza-B. Antibody titres (Anti-SARS-CoV-2-IgG and Anti-SARS-CoV-2-IgA) were positive and SARS-CoV-2-RNA N-gene 1 (PCR) was negative 5 days after primary admission.

The patient had no previously known pulmonary or thoracic diseases. Due to a traumatic motorcycle accident, the patient had undergone splenectomy years before. The patient was under treatment with Dovato^®^ 50/300 mg (GSK^®^, Dolutegravir/Lamivudine) human immunodeficiency virus (HIV) infection, HIV-1-RNA testing (PCR) showed a level of < 40 copies/mL. The patient was treated for COVID-19 pneumonia with supportive measures and discharged with lowering CRP levels as well as normalized leucocytes and interleukine-6 levels after 7 days.

Four days later, 11 days after his initial presentation, the patient was admitted to the emergency department with pronounced dyspnoea after coughing. The clinical and radiographic examination revealed a right-sided tension pneumothorax. To relieve the tension pneumothorax, a small caliber chest tube (20 Charrière = French, Buelau) was inserted through a mini open thoracotomy in the 5th right intercostal space. After an almost complete regression of the pneumothorax the chest tube was removed after 8 days and the patient was discharged after 9 days (20 days after primary admission) in stable condition.

For a period of 3 months after discharge, the patient complained of fatigue with mild joint pain and dyspnoea. After 3 months, the patient reported no residuals of the infection or the tension pneumothorax. During the course of a year, he had regular check-ups including a pulmonary function test, blood tests, X-rays, and computed tomography (CT) scans that are outlined in detail in the following.

The clinical examination 12 months after first admission revealed no persisting symptoms. The surgical wound fully healed without irritation. The soft tissue showed a satisfying aesthetic result, the scar being clean and dry.

After 12 months, the 36-item short-form health survey (SF-36) revealed no limitations in physical functioning (average 100), no role limitations due to physical health (average 100) or due to emotional problems (average 100), no signs of fatigue (average 90), no loss of emotional well-being (average 96) or social functioning (average 100), no indication of pain (average 100) and a good state of general health (average 100) [7].

The Quality of live survey (EQ-5D-5L) also showed no limitation in mobility, self-care, usual activities, pain, and anxiety/depression.

Unfortunately, patient-reported quality of life was not assessed at the time of acute diagnosis.

**Fig. 1:**
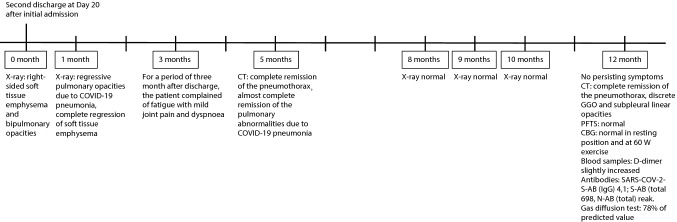
Timeline of the patient history from second discharge to 1 year follow-up. *COVID-19* Coronavirus Disease 2019, *CT* computed tomography, *GGO* ground glass opacities, *SARS-CoV-2* severe acute respiratory syndrome coronavirus 2, *AB* antibodies, *PFTS* pulmonary function tests, *CBG* capillary blood gas

Figure 1 presents the timeline of the events.

## Chest X-rays and CT scans

**Fig. 2:**
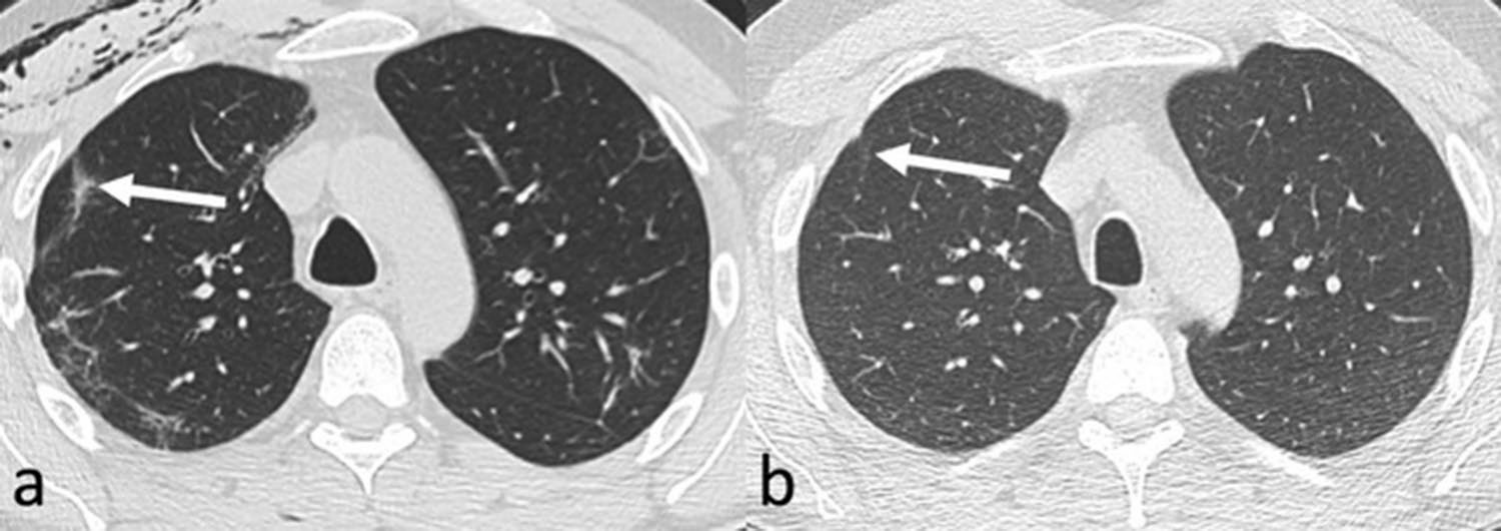
Radiological follow-up after tension pneumothorax due to COVID-19 pneumonia. Thoracic computed tomography performed 1 day before second discharge from the hospital (**a**) shows subpleural linear and reticular consolidations as a sign of COVID-19 pneumonia (arrow). Furthermore, there is right-sided soft tissue emphysema due to the pneumothorax (the latter not shown). One year later (**b**), the pulmonary abnormalities due to COVID-19 pneumonia have almost completely resolved. Only very few, discrete subpleural linear consolidations and GGO (arrow), which presumably correspond to minor post-inflammatory fibrotic changes, are visible. *COVID-19* coronavirus disease 2019, *CT* computed tomography, *GGO* ground-glass opacities

Eight days after second admission, thoracic X-rays showed right-sided soft tissue emphysema and bipulmonary opacities due to COVID-19 pneumonia. The pneumothorax was no longer visible on radiographs. CT, however, showed residuals of the pneumothorax, as well as soft tissue emphysema and mild pneumomediastinum. Furthermore, bipulmonary signs of COVID-19 pneumonia (ground glass opacities (GGO) and consolidations with peripheral distribution) were depicted on CT images at this time point. Thoracic X-ray performed 1 month after second discharge from the hospital showed regressive pulmonary opacities due to COVID-19 pneumonia. Further chest X-rays performed 8, 9, and 10 months after second discharge were all normal. CT scans acquired 5 and 12 months after second discharge showed complete remission of the pneumothorax and almost complete remission of the pulmonary abnormalities due to COVID-19 pneumonia. One year after second discharge only discrete GGO and subpleural linear opacities, presumably corresponding to mild post-inflammatory fibrotic changes, remained detectable on CT images (Fig. 2) [8].

## Clinical course

Pulmonary function tests after 12 months showed a forced vital capacity (FVC) of 103% of predicted value (6360 mL), and a forced expiratory volume in one second (FEV 1) of 109% of predicted value (5240 mL). Capillary blood gas values measured at rest on room air were normal (pH 7.42; pCO2 37.5 mmHg; pO2 80.7 mmHg and SO2 97%) and there was a normal physiological increase in pO2 with light exercise (60 W) resulting in pH 7.41; pO2 86.3 mmHg, pCO2 38.3 mmHg, SO2 97%. The gas diffusion test revealed a minimally decreased diffusion capacity of 78% of predicted value [12.36 mmol/(min*kPa)].

## Blood samples

Initially, in March 2020, laboratory parameters showed increased levels of CRP (10.2 mg/d), lactate dehydrogenase (LDH) (406 U/L), leucocytes (12.4 G/L), D-dimers (1.7 μg/mL) and interleukine- 6 (122 pg/mL).

The blood samples after 12 months revealed normal CRP (0.4 mg/dl), LDH (231U/L), leucocytes 8,49 G/L and Interleukine-6 (5.4 pg/ml). Neutrophil count (41%) and lymphocyte count (45%) were also normal. D-Dimer levels were still increased (0.9 μg/mL) but lowered compared to the D-dimer levels in March 2020.

Tests for SARS-CoV-2 N antibodies (total; Roche) were positive (698U/ml), as were as SARS-CoV-2 S antibodies (IgG; Euroim.) (4:1 ratio).

One month before admission, at a regular check-up, CD4 count was 1408 cells/µl (35% of lymphocytes) and CD4/CD8 ratio 0.9. Nadir CD4 count during the subsequent COVID-19 illness was 573 cells/µl (23% of lymphocytes) and lowest CD4/CD8 ratio 0.47 2 days after hospitalization. Three months after discharge the CD4 count was 1445 cells/µl (57% of lymphocytes) and CD4/CD8 ratio 0.84. One year after discharge the CD4 count was 1195 cells/µl (45% of lymphocytes) and CD4/CD8 ratio 0.91.

## Discussion

Since the beginning of the pandemic in late 2019 little is known about long-term sequelae of COVID-19. Carfi et al. reported that 87% of hospitalized COVID-19 patients (*n* = 143) continued to experience at least one persistent symptom 60 days after onset of the disease, and 44% had worsened quality of life [3]. In another study, Taboada et al. found that 6 months after hospitalization the functional status decreased in 47.5% of the patients. Fumagalli et al. examined the respiratory function at the time of clinical recovery and 6 weeks after discharge in 13 patients surviving COVID-19 pneumonia. Their findings suggest that COVID-19 pneumonia may result in clinically relevant alterations in pulmonary function tests, with a mainly restrictive pattern [9]. In our case, pulmonary function tests after a year were normal without a sign of a restrictive pattern. Gas diffusion test revealed a decrease of the diffusion capacity of 78% of predicted value.

Pneumothoraces in association with COVID-19 infections are still a rare complication. Spontaneous pneumothoraces were described in only 1% of cases, predominantly in male patients (3.3:1) [10, 11]. Delayed pneumothoraces were reported in several cases [12, 13]. While the exact process of the development of a delayed pneumothorax remains unknown, persistent chronic inflammatory changes and delayed alveolar damage due to coughing are discussed as a possible cause [14]. In our case, the patient was admitted to the hospital with pneumothorax after coughing.

Morin et al. presented an uncontrolled cohort study of 478 survivors of COVID-19 4 months after hospitalization. 244 patients (51%) reported at least one new-onset symptom. The most common symptoms were fatigue in 134 of 431 (31%), cognitive impairments in 86 of 416 (21%), and dyspnoea in 78 of 478 (16%) patients. CT lung scan abnormalities were reported in 63% of 171 patients assessed at an ambulatory visit, mainly subtle GGO. Fibrotic lesions were observed in 19% of these 171 patients [2]. These findings are in line with the clinical manifestations of our case. Here the patient showed relevant symptoms for the first 3 months after the infection and secondary tension pneumothorax. One year after hospitalization, the patient was asymptomatic with no loss in quality of life. The CT scan revealed only subtle GGO and subpleural linear opacities, presumably corresponding to mild post-inflammatory fibrotic changes.

Immunological dysfunction such as HIV infection is most likely a risk factor for disease severity [15, 16]. However, this could not be confirmed in a German series of 33 HIV-infected patients with COVID-19 and a series of 26 HIV-infected, COVID-19 diagnosed patients in northern Italy, where morbidity and mortality of COVID-19 were comparable to those reported in other cohorts of HIV-negative patients [17, 18]. In our case, based on the blood samples and the medical history of the patient within the last year, the HIV infection showed no progression with stable CD4/CD8 ratio. Furthermore, blood samples revealed normal levels for CRP, LDH, leucocytes, and Interleukine-6 while the antibody titers were still relatively high.

Townsend et al. reported increased D-dimer levels up to 4 months in 25% of patients after SARS-CoV-2 infection. In our patient, D-dimer levels were still elevated 1 year after Covid-19 [19].

## Conclusion

We present 1 year follow-up of a case of secondary tension pneumothorax as a complication of SARS-CoV-2 infection. After 1 year, the patient did not suffer from any persisting symptoms. The pulmonary function was normal as were the blood parameters except the D-Dimer levels, which remained slightly increased. The CT scan revealed only discrete GGO and subpleural linear opacities. The HIV infection showed no progression.

## Data Availability

All available information is contained within the manuscript.
